# Administration of Fozivudine Tidoxil as a Single-Agent Therapeutic during Acute Feline Immunodeficiency Virus Infection Does Not Alter Chronic Infection

**DOI:** 10.3390/v4060954

**Published:** 2012-06-07

**Authors:** Michelle M. Miller, Jonathan E. Fogle

**Affiliations:** Immunology Program, Department of Population Health and Pathobiology, College of Veterinary Medicine, North Carolina State University, Raleigh, NC 27607, USA; Email: mmdollar@ncsu.edu

**Keywords:** acquired immunodeficiency syndrome, lentivirus, FIV, antiretroviral therapy (ART), Zidovudine, Fozivudine

## Abstract

Initiating combination antiretroviral therapy (ART) during acute HIV infection has been correlated with decreased viral set point and improved lymphocyte function. However, the long term effects of single-agent therapy administered only during the acute stage of infection (interrupted treatment) remain largely uncharacterized. In this study we provide longitudinal data using the feline immunodeficiency virus (FIV) model for HIV infection. Infected cats were treated with a prophylactic single-agent therapy, Fozivudine tidoxil (FZD), for six weeks, starting one day before infection. The initial acute infection study, reported elsewhere, demonstrated a decrease in plasma- and cell-associated viremia at two weeks post-infection (PI) in FZD-treated cats as compared to placebo-treated cats. We hypothesized that this early alteration in plasma- and cell-associated viremia would alter the virus set point and ultimately affect the outcome of chronic infection. Here we provide data at one, two and three years PI for plasma- and/or cell-associated viremia, total lymphocyte counts and CD4:CD8 ratios. There was no difference in viremia or cell counts between treated and nontreated groups at all time points tested. Contrary to our hypothesis, these results suggest that treatment with a single agent anti-retroviral drug during acute lentivirus infection does not significantly alter viral load and immune function during the chronic, asymptomatic stage of infection.

## 1. Introduction

Although there is not uniform agreement on when (and if) to initiate early treatment of HIV patients with anti-retroviral therapy (ART), evidence suggests early therapy correlates to improved immune responses and reduced viral replication during the course of infection [1,2,3,4]. The protective effect of early drug therapy may result from decreased viral set point as several studies have demonstrated that stable, high levels of virus during chronic infection are associated with more rapid disease progression than that observed for patients maintaining low viral set points [5,6,7]. Delayed seroconversion with early ART treatment has also been described [8]. These studies use various combination therapies, typically administered over long periods of time with only short term disruptions in treatments [1,2,3,4,5,6,7].

Little is known regarding the long term effect of single therapeutic treatment, administered only during acute infection, on immune function and virus load during the later chronic stage. The timing of infection and ethical considerations of altering or withholding treatment make this a difficult question to address in HIV infected patients. Using the Feline Immunodeficiency virus (FIV) model for AIDS lentiviral infection, we hypothesized that treatment with a single agent antiviral therapy, Fozivudine tidoxil (FZD), prior to and during the acute stage of FIV infection would decrease acute viremia, thus altering the virus set point as chronic infection is established. FZD administration was stopped after six weeks and the cats were followed longitudinally for three years post-infection (PI). The initial study is reported elsewhere and demonstrated a decrease in plasma and cell associated viremia at two weeks PI in FZD-treated cats as compared to placebo-treated cats [9].

FZD is related to the commonly used HIV therapeutic, Zidovudine (ZDV, also known as AZT). Both are members of the NRTI family of drugs which are incorporated into the newly synthesized strand of DNA during intracellular viral replication and irreversibly bind viral RT which disrupts viral reverse-transcription [10]. FZD is a thioether lipid-ZDV conjugate. The lipid portion is removed following intracellular cleavage and the remaining ZDV monophosphate is phosphorylated into the active form of the drug [11,12]. This requirement for intracellular processing decreases the potential for hematologic toxicity and reduces the concern for anemia in treating FIV infected cats [11].

Most studies utilizing ZDV in the feline model have focused on acutely infected and chronically infected cats as isolated events. There are a handful of studies providing longitudinal data on experimentally infected cats, however; these studies do not report data past the first year of infection, limiting our knowledge on long term effects of such treatments [13,14,15,16]. Here, we provide data up to three years PI to evaluate any long-term effects of prophylactic FZD treatment. Plasma viremia was measured at one, two and three years PI and cell-associated viremia was measured at three years PI. Total lymphocyte counts, CD4:CD8 ratios and hematocrit values were also analyzed at two and three years PI.

## 2. Results and Discussion

### 2.1. Plasma and Cell Associated Viremia

Plasma viremia was assessed at one, two or three years PI by real-time RT-PCR for the placebo-treated and FZD-treated cats (Table 1). All cats had detectable virus regardless of treatment. Mean viremia was calculated and no significant differences were found between treatment groups for each time point tested. At three years PI, cell-associated viremia was also assessed by real-time RT-PCR for placebo-treated and FZD-treated cats. Only two out of five (40%) of the FZD-treated cats had detectable cell-associated viremia compared to three out of five (60%) of the placebo treated cats. No significant differences in the mean cell-associated viremia were found between treatment groups.

**Table 1:** Assessment of plasma- and cell-associated viremia in Fozivudine tidoxil (FZD) and placebo-treated cats during chronic Feline Immunodeficiency virus (FIV) infection. The percentage of positive cats by PCR analysis is shown in the first row for FZD- and placebo-treated groups and the corresponding number of cats out of each group is displayed on the second row. The mean, median, and range of the plasma viremia in number of virus copies/mL are listed for each treatment group at 1, 2, and 3 years PI. The mean, median and range of the cell-associated viremia in copies/10^6^ Peripheral blood mononuclear cells (PBMCs) are listed for each treatment group at 3 years PI. Five cats were evaluated for each experimental group. (*p* < 0.05, NS = not significant).

	FIV-gag-mRNA			
	[copies/mL plasma]			[copies/10^6^ PBMCs]
Years Post infection	1	2	3	3
**FZD**				
Positive (%)	100	100	100	40
	(5/5)	(5/5)	(5/5)	(2/5)
Mean	2.91	4.84	2.74	1.44
Median	2.80	4.26	2.77	1.44
Range	2.27–3.67	3.87–6.76	2.04–3.85	1.41–1.46
**Placebo**				
Positive (%)	100	100	100	60
	(5/5)	(5/5)	(5/5)	(3/5)
Mean	3.40	4.81	2.59	1.39
Median	3.32	4.27	2.33	1.46
Range	2.96–3.98	3.89–6.81	1.93–3.97	1.16–1.54
***p-*value**	NS	NS	NS	NS

### 2.2. Lymphocyte Enumeration and Composition

**Figure 1:**
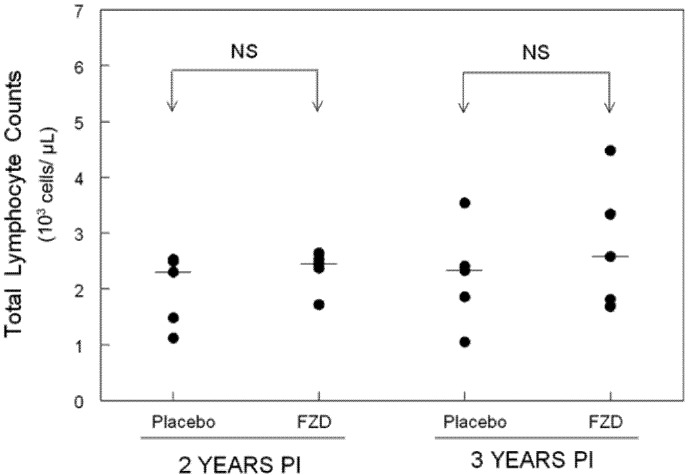
Total lymphocyte counts in placebo- and FZD-treated cats during chronic FIV infection. No statistical differences in total lymphocyte counts were observed between Fozivudine (FZD)-treated cats and placebo-treated cats at two or three years PI. The mean lymphocyte count is displayed as a line for each group evaluated.Five cats were evaluated for each experimental group. (p < 0.05, NS = not significant).

**Figure 2:**
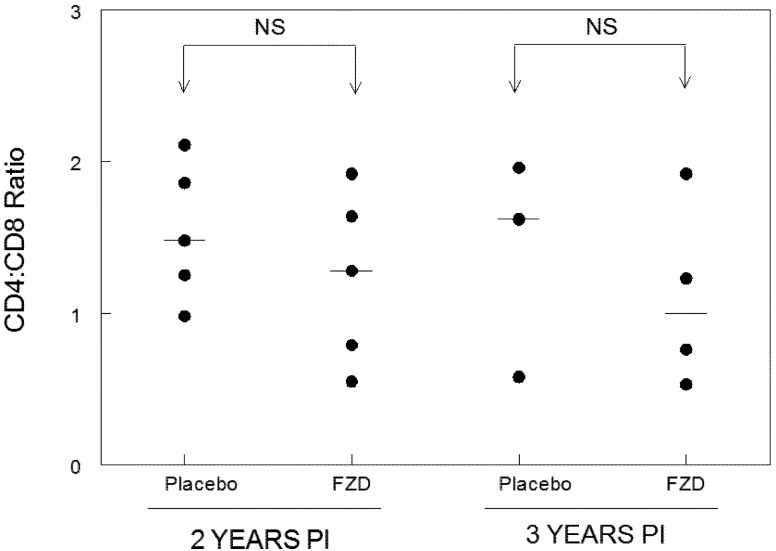
CD4:CD8 ratios in placebo- and FZD-treated cats during chronic FIV infection. No statistical differences in CD4:CD8 ratios were observed between Fozivudine (FZD)-treated cats and placebo-treated cats at two or three years PI. The mean CD4 to CD8 ratio is displayed as a line for each group evaluated. Five cats were evaluated for each experimental group at two years PI. Three placebo-treated and four FZD-treated cats were analyzed at three years PI. (*p* < 0.05, NS = not significant).

Total lymphocyte counts were performed at two and three years PI for each group (Figure 1). Total lymphocyte counts were not statistically different between treatment groups and values remained stable between time points. Flow cytometry analysis was also performed on PBMCs from each cat at two and three years PI to assess lymphocyte composition and to calculate the CD4:CD8 ratio (Figure 2). The CD4:CD8 ratio did not differ between FZD-treated and placebo-treated groups.

### 2.3. Clinical Findings

The cats were observed daily for changes in attitude and appetite by laboratory animal resource personnel. Routine physical examinations were performed by a veterinarian or certified veterinary technician during each sample collection. Aside from sporadic, isolated, mild gingivitis/stomatitis, physical examination of all cats was unremarkable. As no observable outbreaks of disease occurred in this group of cats and because strict SPF protocol was observed, the cats reported here were not tested for other pathogens. The mean hematocrit values were assessed at years two and three PI and remained between 30 and 35% with no significant differences between FZD-treated and placebo-treated groups. All other RBC indices tested were within normal ranges and did not differ significantly between groups.

## 3. Experimental Section

### 3.1. Cats

Specific pathogen-free cats were obtained from a commercial vendor at 6 months of age and housed in the Laboratory Animal Resource Facility at the College of Veterinary Medicine, North Carolina State University. Cats were maintained in isolated SPF conditions from time of receipt. The initial report on Fozivudine treatment utilized 6 FZD-treated cats and 6 placebo control cats (9). For the follow-up study reported here, 5 cats from each group were available for assessment as outlined in Table 1 and Figure 1. For Figure 2, 5 cats were available for lymphocyte analysis at 2 years PI and 3 placebo cats and 4 FZD cats were available for lymphocyte analysis at 3 years PI (several had been sacrificed for unrelated studies). Protocols were approved by the North Carolina State University Institutional Animal Care and Use Committee.

### 3.2. Fozivudine Tidoxil

(HDP 99.0002) FZD tidoxil (3'-azido-3'-deoxy-5'-thymidylic acid, mono[3-(dodecylthio)-2-deoxy-decycloxypropyl] ester, sodium salt) is a ZDV-thioether lipid conjugate. It is a white to off-white power which should be stored between 15 and 30 °C. For these experiments, FZD was prepared in either 170 or 187 mg gelatin capsules for administration PO at an average dosage of 45 mg/kg twice daily (90 mg/kg/d). The dosage range was 42–46 mg/kg twice daily. Placebo consisted of equal amount of sucrose prepared in gelatin capsules. All gelatin capsules were prepared at a compounding facility and shipped to the NCSU-CVM. FZD or placebo administration was randomized and non‑blinded. FZD or placebo was administered starting one day before FIV challenge for a total of six weeks and then discontinued. No additional treatments were performed. At the time of administration, the use of FZD tidoxil was licensed to Piedmont Pharmaceuticals (Greensboro, NC, USA) by Heidelberg Pharma AG (Ladenburg, Germany).

### 3.3. Infection with FIV

The NCSU_1_ isolate of FIV was originally obtained from a naturally infected cat at the North Carolina State University College of Veterinary Medicine and has been described in detail elsewhere [17]. Virus inoculum was grown as a single-tissue culture passage in an IL-2-dependent feline CD4+ cell line (FCD4-E cells) as described previously [18]. The cats were inoculated IV with 5 × 10^5^ TCID_50_ of cell-free virus culture. The cats used for the previously reported acute study were infected at 7 months of age then maintained and housed at the NCSU-CVM as described above [9]. No additional treatments or medications were administered to any cats prior to sample collection for the current study.

### 3.4. Sample Collection

Blood was collected at one, two or three years following initial infection. Blood was collected by jugular venipuncture into EDTA vacutainer tubes and 2 mL were retained for a CBC and lymphocyte subset analysis by multicolor flow cytometry. Plasma was separated from the remaining blood and frozen (−20 °C) until analysis of viral load by real time RT-PCR.

### 3.5. Lymphocyte Subset Analysis

The phenotype of lymphocytes from peripheral blood was determined by multicolor flow cytometric analysis. Peripheral blood mononuclear cells (PBMCs) were stained with anti-CD4-Strepavidin/PerCP and anti-CD8-PE by an established whole blood lysis protocol [18]. The murine monoclonal antifeline CD4 (mAb 30A) and CD8 (mAb 3.357) used to stain PBMCs were produced in the author’s laboratory [19]. For flow cytometric analysis, lymphocytes were gated based on forward versus side scatter and approximately 20,000 gated events were acquired and stored list-mode fashion for analysis by CellQuest software. Five cats were analyzed for each treatment group at two years PI. We were unable to perform lymphocyte subset analysis on all cats at three years PI as two placebo-treated and one FZD-treated cats were sacrificed for unrelated studies.

### 3.6. Plasma and Cell-Associated Viremia

Quantitative real-time PCR was used to determine viral gag-mRNA loads in each plasma sample and was reported as copies/mL plasma. PCR quantification of FIV gag was performed using FIV gag standards of known concentrations in order to determine copy numbers. This assay can detect FIV gag as low as 10^−2^ copies/mL or 10^−2^ copies/10^6^ cells which is below any of the plasma or cell-associated viremia values reported here. PBMCs were isolated by Percoll density gradient centrifugation and 1 × 10^6^ PBMCs were then stored in RNA Protect (Qiagen) until analysis. The primer and probe sequences used for FIV-gag have been reported elsewhere [20,21].

### 3.7. Statistical Analysis

Cats with undetectable cell-associated viremia were not included in calculations of mean or median (Table 1). A Mann-Whitney U-test was used to compare viremia, total lymphocyte counts and hematocrit from FZD-treated and placebo-treated cats with *p* < 0.05 considered significant.

## 4. Conclusions

Evidence suggests that anti-retroviral therapy during early acute HIV will alter dynamics of chronic infection. Longitudinal studies on patients receiving ART have additionally shown that administration of combination drugs prolongs survival, enhances immune responses and delays onset of AIDS [1,2,3,4]. The protective effect of early drug therapy may be a result of decreased viral set point and delayed viremia, as several studies have demonstrated that high viremia during chronic infection can be indicative of more rapid disease progression when compared to patients which maintain a low viral set point [5,6,7]. Taken together, these studies suggest that administration of therapeutic drugs during early infection may reduce morbidity associated with chronic immune activation, slow progression to AIDS, and increase patient survival times.

The cats in the current study were initially part of a non-blinded, placebo controlled study to assess the effectiveness of a novel nucleoside analogue reverse transcriptase inhibitor (NRTI), FZD, during acute FIV infection [9]. The results of this study showed that FZD was effective at lowering plasma- and cell-associated viremia at two weeks post-FIV infection with a trend toward lower plasma- and cell- associated viremia at four and six weeks PI. Additionally, lympholysis associated with acute viremia was noted in the placebo-treated group but not in the FZD-treated group during acute infection suggesting a protective effect of drug therapy [9]. The results from this acute infection study suggested that early treatment with only one anti-viral drug may alter the virus set point and therefore the outcome of chronic infection. We hypothesized that the FZD-treated group as compared to the placebo-treated group would exhibit lower plasma viremia and higher lymphocyte counts during the chronic stages of infection. In a similar study using ZDV as a single agent therapy, Hayes *et al.* [14] treated experimentally infected cats with ZDV 48 hours prior to FIV infection and continued treatment for a total of 28 days. After 28 days, treatment was discontinued and the cats were followed for 1 year PI. In this study, the ZDV-treated cats exhibited a dramatic reduction in FIV antigenemia during the first 4 weeks of therapy as compared to untreated FIV-infected controls. However, they were unable to document a difference in plasma antigenemia between ZDV-treated and untreated cats during the chronic stage of infection. In the study presented here, we were able to follow cats for 3 years PI. Although FZD treatment during the acute stage of infection lowered the initial viremia, the results of our analysis suggest there was no effect on plasma viremia from 1–3 years PI and no effect on cell-associated viremia at 3 years PI, similar to the findings reported by Hayes *et al* [14,15]. Interestingly, Hayes *et al* were able to document improvement in CD4:CD8 ratios in ZDV-treated cats consistently out to 1 year PI. In the current study, total lymphocyte counts and CD4:CD8 ratios were evaluated at one, two or three years PI and no significant differences were found between treatment groups. We hypothesized that lowering the initial virus burden may reduce immune activation and therefore improve CD4:CD8 ratios. However, as reported in our initial study, there was no difference in CD4:CD8 ratios between treatment groups during the acute stage of infection and this trend appeared to continue into the chronic stage of infection. Taken together, the results of the current study indicate that prophylactic FZD treatment followed by discontinuation of therapy has no effect on virus set-point as measured by plasma- and cell-associated viremia and no long term effect on CD4:CD8 lymphocyte ratios.
